# Predictors of time to recovery from uncomplicated severe acute malnutrition among children in eastern Ethiopia

**DOI:** 10.3389/fnut.2024.1275943

**Published:** 2024-06-06

**Authors:** Shibiru Kelbessa Yadeta, Trhas Tadesse, Tarekegn Negese, Bisrat Haile, Aweke Kebede, Aboma Motuma, Dureti Abdurahman, Abdu Oumer, Kedir Teji Roba

**Affiliations:** ^1^National Nutrition Program Officer, Nutrition Coordination Office in the Ministry of Health, Addis Ababa, Ethiopia; ^2^Department of Social and Population Health, Yekatit 12 Hospital Medical College, Addis Ababa, Ethiopia; ^3^Monitoring and Evaluation Officer in the Nutrition Coordination Office, Ministry of Health, Addis Ababa, Ethiopia; ^4^Implementation Advisor, Nutrition Coordination Office in the Ministry of Health, Addis Ababa, Ethiopia; ^5^World Food Program, Addis Ababa, Ethiopia; ^6^School of Public Health, College of Health and Medical Sciences, Haramaya University, Harar, Ethiopia

**Keywords:** outpatient therapeutic program, time to recovery, severe acute malnutrition, treatment outcome, Ethiopia

## Abstract

**Background:**

Managing severe acute malnutrition (SAM) involves an outpatient therapeutic program (OTP), targeting more than 80% of SAM children where the quality of primary healthcare remains poor. Treatment success and recovery from SAM remain poor and could be affected by many factors, where such evidence is limited in East Hararghe. This study assessed the predictors of time to recovery from SAM in eastern Ethiopia.

**Methods:**

A retrospective cohort study was conducted on 402 records of SAM children under 5 years of age enrolled on OTP at 12 health posts retrieved from 2020 to 2021. We used the Kaplan–Meir estimate along with the *p*-value of the log-rank test and the survival curve to compare the time to recovery across categories. A multivariable Cox proportional hazard model was fitted to identify predictors of time to recovery from SAM. A *p*-value below 0.05 was used to declare statistical significance.

**Results:**

A total of 402 records were reviewed, and the cure rate from SAM was 89.6% [95% confidence interval (CI), 87–93]. Moreover, a death rate of 0.7%, a default rate of 9.5%, and a non-responder rate of 0.2% were obtained with a median length of stay of 7 weeks. The median time to recovery was significantly shorter for children from shorter distances from OTP sites with edema, amoxicillin, (*p* < 0.05). Edema at admission [adjusted hazard ratio (AHR) = 1.74; 95% CI: 1.33–2.29], without diarrhea (AHR = 1.51; 95% CI: 1.18–1.94), taking amoxicillin (AHR = 1.55; 95% CI: 1.19–2.02), shorter travel time to the OTP site (AHR = 1.44; 95% CI: 1.13–1.85), breastfeeding (AHR = 1.60; 95% CI: 1.27–2.02), adequacy of ready-to-use therapeutic food (RUTF) (AHR = 1.22; 95% CI: 0.90–1.65), and new admission (AHR = 1.62; 95% CI: 0.84–3.10) were important predictors of recovery from SAM.

**Conclusion:**

Recovery from SAM was found to be acceptable in comparison with the Sphere Standards and is predicted by edema, diarrhea, distance from the OTP site, amoxicillin, and RUTF adequacy. These allow for focused interventions that address the identified factors for better recovery from SAM.

## Introduction

Acute malnutrition develops over a short period of time in response to inadequate dietary intake and infections (1), which is graded as moderate and severe acute malnutrition (SAM). SAM is defined as a mid-upper arm circumference < 115 mm, or a weight-for-height/length < −3 Z-score of the World Health Organization (WHO) child growth standards medians, or bilateral pitting edema (2). Every year, approximately 45.5 million children are affected by acute malnutrition, and 13 million are severely malnourished (3). In Africa, almost 13.8 million under-five children were acutely malnourished (4). Moreover, it leads to 1.7 million child deaths annually in sub-Saharan Africa (2), increasing the risk of mortality by nine-fold (5, 6). These figures have increased by 6.7 million due to the current COVID-19 pandemic in recent years (7, 8). In particular in developing countries, more than 2% of children are severely malnourished (9). The recent national survey of Ethiopia has also shown that 11% of children were wasted in Ethiopia, and the prevalence was 9% in the Oromia region (10), indicating a rise in recent years.

Malnutrition is a major cause of morbidity, mortality, and long-term developmental deficits. For instance, approximately 45 percent of children under the age of five die as a result of malnutrition (8), and children who are wasted are at risk for long-term developmental deficits (2). Compared to the global target to reduce wasting below 5%, there is an alarming rate of wasting aggravated by natural and man-made disasters. Ethiopia has been hit by a series of natural and manmade disasters, most notably the cyclic drought, which has had a severe influence on the efforts of the country to reduce acute malnutrition (11, 12). Despite the fact that the magnitude of wasting has declined from 13 to 7% from 2016 to 2019, the burden of SAM in the country is increasing alarmingly, coupled with the prevailing civil war, rising food prices, and others (13). Hence, acute malnutrition contributes to a total loss of US $230 million and up to 64% of the economic burden (14).

The Community Management of Acute Malnutrition (CMAM) is found to be effective for the rapid recovery of SAM children and the prevention of future crises. However, the coverage is still approximately 56%, as reported in 2020, where a significant number of SAM children are being missed (15). The outpatient therapeutic program (OTP) had a higher coverage (79–95% of SAM children) for children suffering from uncomplicated SAM to recover (16), although treatment success is limited by various factors that need to be investigated. A study showed a high recovery rate of 92%, low mortality of 0.1%, and acceptable default rates (7.5%) among SAM children. The implementation of CMAM has increased access to malnutrition care closer to home and significantly reduced deaths. However, only approximately 20% of children with SAM receive treatment at a facility (17), where the OTP makes it easier to treat children at home with maximum coverage. The program targets SAM children above 6 months without medical complications or transfer outs from inpatient care for better coverage and treatment success (18, 19). The results of treatment are categorized as cured, defaulted, died, and non-respondent to treatment, which are used as indicators for treatment success (20).

The Sphere Humanitarian Standards is a widely recognized set of guidelines and principles for humanitarian response in emergency settings. These standards provide a framework for improving the quality, effectiveness, and accountability of humanitarian interventions. In the context of recovery from severe acute malnutrition (SAM), adherence to the Sphere Humanitarian Standards becomes crucial in ensuring that interventions meet minimum benchmarks and prioritize the wellbeing and rights of affected individuals (21, 22). This allows us to understand the specific needs and challenges associated with SAM recovery, leading to more effective and coordinated interventions. The standard sets more than 75% recovery, less than 10% death, and less than 15% default rates (21).

Although poor treatment outcomes could be lower and previous studies focused on inpatient care, there is limited evidence to elucidate treatment success and potential factors limiting optimal treatment outcomes. In addition, the OTP targets 80% of the SAM children, where evaluation of program success is vital for policy decisions (5, 23). East Hararghe faces numerous nutritional challenges, worsened by high fertility rates and low agricultural productivity. However, there is a lack of evidence for understanding the factors influencing the time it takes for individuals to recover from SAM, which is crucial for addressing the existing situation. This study aimed to fill this knowledge gap by providing valuable evidence specific to the context of East Hararghe, where SAM occurrence is prevalent but such evidence is lacking. The objective of this study was to assess the significant factors that predict the time to recovery from uncomplicated SAM in the context of the OTP in eastern Ethiopia.

## Materials and methods

### Study area and period

East Hararghe zone is one of the 20 Zones in the Oromia region, located in the eastern part of Ethiopia. The zone has 20 rural and 4 urban woredas with 543 kebeles (the least administrative). East Hararghe zone is located 510 km to the east of Addis Ababa, Ethiopia. The zone has a total population of 4.04 million. The zone has 557 health posts, 121 health centers, and 8 hospitals; these facilities make up the health service coverage of the zone (24). From the total population, under 5-year-old children were 682,796 and the zone has approximately 650 OTP sites. It is one of the most populous zones in the region. In addition, it is among the chronically food-insecure areas of the country that face recurrent drought and poverty. The data were collected from 20 May 2022 to 20 June 2022 whereas the retrieval period was from 2020 to 2022.

### Study design and population

An institution-based retrospective cohort study was employed among SAM children in the OTP program via chart review. The current study targeted records of uncomplicated SAM children aged from 6 to 59 months who were on OTP treatment between 8 July 2020 and 7 July 2021. Those children admitted according to the recent national SAM protocol were included (MUAC value 11.5 cm or bilateral pitting nutritional edema, good appetite test, and no medical complications). Records of SAM children referred for inpatient care against medical advice were excluded. In addition, records of children with incomplete outcome variables and missing more than 50% of the independent variables were dropped.

### Sample size determination and sampling methods

A two-population proportion sample size estimation formula was employed in Epi Info software. We considered 95% confidence level, 80% power, 5% margin of error, a ratio of unexposed to exposed of 1:1, the proportion of children with diarrhea who were recovered (exposed group) = 47.4%, and the proportion of children without diarrhea who were recovered (non-exposed group) = 65.9% (25). Furthermore, we assumed a design effect of 1.5 to account for heterogeneity and a 10% card withdrawal rate; a total of 402 SAM records were required for this study.

We employed stratified random sampling with proportional allocation to select samples for four selected districts of the eastern Hararghe zone. Then, three OTP sites were randomly selected from each district, for a total of 12 OTP sites. Finally, the individual SAM records from each selected OTP site were selected using systematic random sampling at each sample interval based on their unique medical record number and/or SAM registration number. The number of medical records from each of the OTP sites was selected proportionally to the total number of SAM records at each OTP site. Then, the allocated number of SAM records was known, and each medical record was selected at every sampling interval. The starting sample record was selected randomly, and the sample at each sample interval was selected accordingly (26). Children aged 6–59 months on OTP from 8 July 2020 to 7 July 2021 in four woredas were 8,850. These numbers were allocated according to their population size. Hence, 143 (35.6%) of the sample was from Fadis, 114 (28.4%) from Kersa, 98 (24.4%) from Haramaya, and 47 (11.7%) from Kombolcha districts.

### Variables of the study

The outcome variable was time to recovery from SAM as defined as recovery from acute malnutrition as per the national and WHO SAM protocols. Recovery from SAM is determined based on significant weight gain, improvement in anthropometric measurements (MUAC in centimeters), resolution of nutritional edema, WFH percentage of the median above 80% and clinical improvement with the absence of edema, improved appetite, and overall enhancement of health and wellbeing (5). This has been tracked from the SAM register and/or the medical chart of children, which is being implemented as per the national SAM guideline, which is a very valid means.

Recovery was coded as recovered and not recovered. The length of time (in days) was extracted from the day of admission and the last recorded follow-up period. Moreover, default is declared when the child is lost to follow-up, and death is recorded when the child dies during the follow-up period (23). Discharge from the OTP program refers to children with SAM who exit the program through cure, death, default, or being non-responders. Cure or recovery was when MUAC was 12.5 cm and the child had no edema for at least 2 weeks, while death was when the child reported death during treatment in outpatient care (1, 5, 27). Default is defined as being absent for two consecutive visits (2 weeks), and non-responders are when the child did not recover after 4 months (16 weeks) of standard OTP care. Finally, transferred-out is when the condition of the child has deteriorated or is not responding to treatment and is referred for treatment in inpatient care or to another OTP facility.

The outcomes were measured as follows;


$$\mathrm{Recovery}\;\mathrm{rate}\;\left(\%\right)=\frac{\begin{array}{l} \mathrm{number}\;\mathrm{of}\;SAM\;\mathrm{children}\;\\ \mathrm{recovered}\;\mathrm{or}\;\mathrm{cured} \end{array}}{\begin{array}{l} \mathrm{total}\;\mathrm{number}\;\mathrm{of}\;SAM\;\\ \mathrm{children}\;\mathrm{on}\;\mathrm{follow}\;\mathrm{up} \end{array}}*100$$

$$\begin{array}{l} \mathrm{Defaulter}\;\\ \mathrm{rate}\;\left(\%\right)=\frac{\mathrm{number}\;\mathrm{of}\;SAM\;\mathrm{children}\;\mathrm{defaulted}}{\mathrm{totalumber}\;\mathrm{of}\;SAM\;\mathrm{children}\;\mathrm{on}\;\mathrm{follow}\;\mathrm{up}}*100 \end{array}$$

$$\mathrm{Death}\;\mathrm{rate}\;\left(\%\right)=\frac{\mathrm{number}\;\mathrm{of}\;SAM\;\mathrm{children}\;\mathrm{died}}{\mathrm{totalumber}\;\mathrm{of}\;SAM\;\mathrm{children}\;\mathrm{on}\;\mathrm{follow}\;\mathrm{up}}*100$$

$$\begin{array}{l} \mathrm{Average}\;\mathrm{length}\;\mathrm{of}\;\mathrm{stay}\;\mathrm{in}\;\mathrm{days}=\mathrm{Date}\;\mathrm{ofdischarge}\;\mathrm{or}\;\mathrm{outcome}-\\ \;\mathrm{date}\;\mathrm{of}\;\mathrm{admission} \end{array}$$

The independent variables included age in months, sex, time taken from OTP sites to the child’s home (in minutes), type of malnutrition, breastfeeding, and provision of amoxicillin at admission; an appetite test at admission; admission weight in kilogram; MUAC on admission in centimeter; edema; and routine medication intakes.

### Data collection procedures

To capture the data, we designed a structured abstraction checklist from the OTP card and nutrition registration logbook by four trained health professionals. We checked the tool on a random sample of 10 records, and the necessary changes were made before data collection. A pretest was conducted on 20 SAM records before implementing the study on a large scale. Extensive and practical training was given to data collectors on the practical ways of extracting and cross-validating data sources. The completed questionnaire and the corresponding patient card were collected daily using an offline data collection tool named Open Data Kit (ODK) and checked for completeness and consistency. Cleaning was done on a daily basis, and data collectors received daily supervision with on-the-spot feedback.

### Data management and analyses

The captured data were checked and cleaned for any inconsistencies and analyzed in SPSS version 20 (28). The data were described and summarized using frequency, percent, and a measure of central tendency. The outcome variable was dichotomized as “cured” and “censored” for the final survival and Cox regression analysis, where the outcome was ascertained at discharge or discharge against medical advice. We employed a life table to explain the patterns of recovery among SAM children. Relevant hazards and survival curves were plotted and interpreted, along with mean or median survival times. To compare survival time among different categories, a log-rank test with its corresponding *p*-value was reported. The time to development of the outcome (cure vs. censor) was calculated from the date of discharge or outcome and the date of admission to the OTP program and expressed in days. We used bivariable and multivariable Cox proportional hazard models to identify factors predicting time to recovery from uncomplicated SAM. The proportional hazard assumption for the Cox proportional hazard model was validated by plotting the log-minus-log survival plot against time by categorical variables and performing a global test using Stata software under the null hypothesis of proportional hazards. Predictors with a *p*-value below 0.25 in bivariable analysis and biologically plausible predictors from previous studies were candidates for the final model (29). Hence, we have considered edema, referral system, and chest indrawing as potentially plausible risk factors for recovery from SAM and considered them for the final model. Adjusted hazard ratios (AHR) with a 95% confidence interval were reported, at which statistical significance was declared at a *p*-value less than 0.05. Interaction and multicollinearity effects were also explored using appropriate statistical approaches.

### Ethical considerations

Formal ethical approval was obtained from the Ethical Review Committee of Addis Ababa Medical and Business College (AAMBC), faculty of postgraduate studies with reference no. of AMBC/stu/10746/14 and dated 26/07/2014EC. A formal support letter was written to the Oromia Health Bureau to facilitate the conduct of the study. The bureau rechecked the ethical compatibility, and the study was implemented. We have obtained written consent from each health facility, and no personal identifiers were collected for this study. As the current study was based on a secondary chart review, obtaining consent from a mother or caregiver was not feasible. In addition, the collected data were kept private with the investigators and will be shared anonymously.

## Results

### Baseline characteristics of SAM children

A total of 402 records of SAM children were included in this study. Approximately 211 (52.5%) were female children, and 288 (71.6%) of them were under 2 years of age. A total of 214 (53.2%) of them were breastfeeding, and 256 (63.7%) of the children took less than 30 min to reach health facilities, whereas 146 (36.3%) took more than 30 min. Almost all 390 (97%) of them were new admissions to the OTP program (Table 1).

**Table 1:** Socio-demographic and admission characteristics of SAM children on treatment at OTP, East Hararghe zone, Oromia Ethiopia, from 8 July 2020 to 7 July 2021 (*n* = 402).

Child characteristics	Category	Frequency	Percent
Sex	Male	191	47.5
	Female	211	52.5
Age	<24 months	288	71.6
	24–59 months	114	28.4
Breastfeeding status	Yes	214	53.2
	No	188	46.8
Admission status	New	390	97.0
	Readmission	3	0.8
	Return after default	9	2.2
Distance (time of travel in minutes)	< 30 min	256	63.7
	> = 30 min	146	36.3
Selected districts	Haramaya	98	24.4
	Fadis	143	35.6
	Kersa	114	28.4
	Kombolcha	47	11.7

Regarding admission characteristics, children were mainly admitted due to MUAC (66.2%) and edema (31.6%). On the other hand, 275 (68.4%) and 113 (28.1%) of children had marasmus and kwashiorkor at admission, respectively. With regard to common co-morbidities, 148 (37%) had at least one medical comorbidity, of which diarrhea (36.8%) and cough (2%) were common. We found that ready-to-use therapeutic food (RUTF) was administered according to the weight of children, and 338 (84.1%) got the recommended RUTF as per the national SAM protocol. Routine medications were also provided at OTP for children in the OTP program. Approximately 298 (74.1%) children have taken amoxicillin, and 310 (77.1%) children have had deworming (Table 2).

**Table 2:** Forms of malnutrition and major comorbidities at admission among SAM children attended OTP service in East Hararghe Zone from 8 July 2020 to 7 July 2021 (*n* = 402).

Variables	Categories	Frequencies	%
Type of malnutrition	Marasmus	275	68.4
	Kwashiorkor	113	28.1
	Marasmic kwashiorkor	14	3.5
Presence of Edema	Yes	127	31.4
	No	275	68.6
Adequacy of RUTF	Adequate	338	84.1
	Not adequate	64	15.9
Presence of diarrhea	Yes	148	36.8
	No	254	63.2
Presence of Cough	Yes	8	2.0
	No	394	98.0
Appetite test done	Good	367	91.3
	Poor	35	8.7
Mebendazole/Albendazole on second visits at admission	Yes	310	77.1
	No	92	22.9
Provision of Amoxicillin at admission	Yes	298	74.1
	No	104	25.9

### Treatment outcomes of SAM children

Of 402 SAM children on OTP service delivery sites, 360 (89.6%) were recovered or cured. In addition, 38 (9.5%) were defaulters, 3 (0.7%) died, and 1 (0.2%) were non-responders among the study participants. The median length of stay at OTP was approximately 7 weeks. Yet, this standard is mainly for emergency settings where further moves are expected to improve good treatment outcomes. More importantly, the median length of stay at the hospital was relatively longer (>6 weeks) (Table 3).

**Table 3:** Performance indicators of OTP and comparison to the Sphere Standards for treatment success among children in East Hararghe Zone, Ethiopia (*n* = 402).

Performance indicators	Frequency of indicators	International sphere standards reference	
		Acceptable	Alarming
Recovery rate (%)	89.6% (*n* = 360)	>75%	<50%
Defaulter rate (%)	9.5% (*n* = 38)	<15%	>25%
Death rate (%)	0.7% (*n* = 3)	<10%	>15%
Average length of stay in weeks		<4 weeks	>6 weeks
<4 weeks	3 (0.7%)	Acceptable	
4–6 weeks	183 (45.5)	Long	
>6 week	216 (53.8%)		Alarming

### Time to recovery from SAM

**Figure 1:**
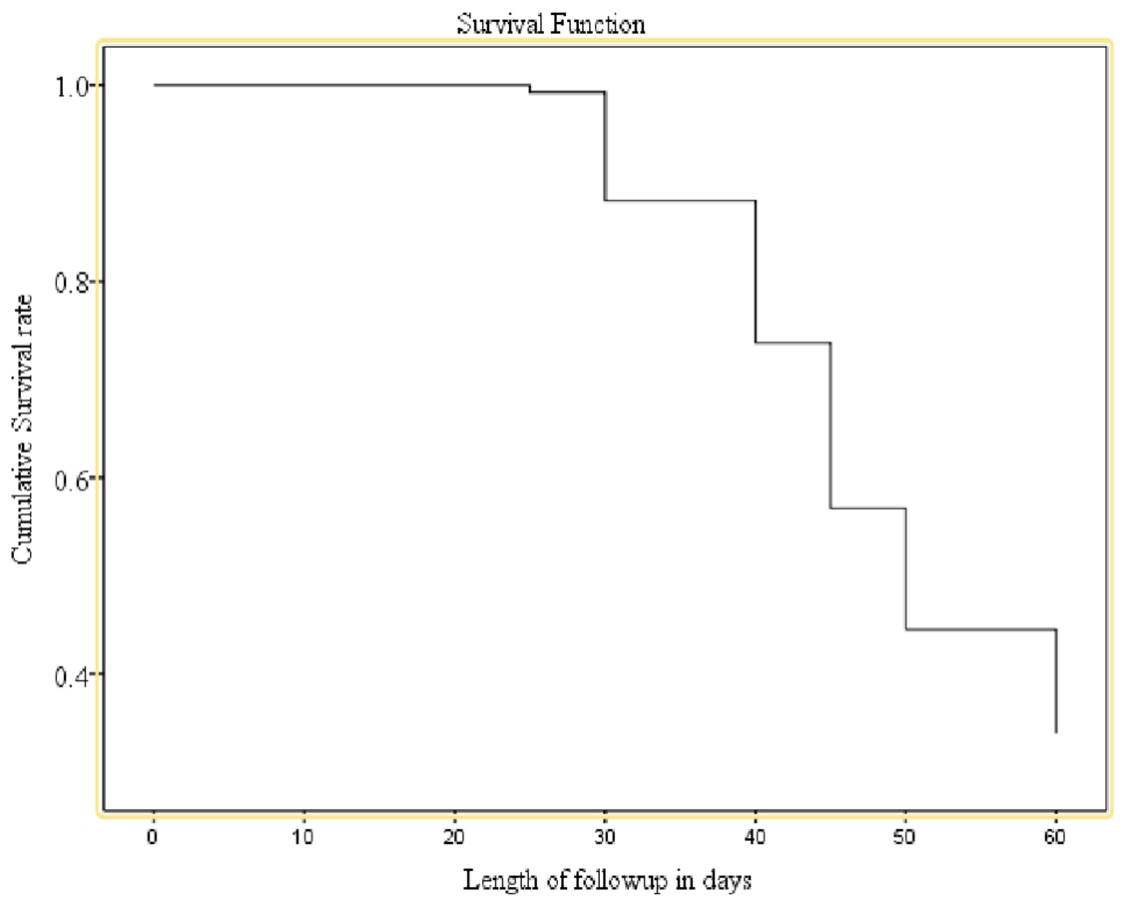
Cumulative survival curve for time to recovery from uncomplicated SAM, in eastern Ethiopia.

Approximately 89.6% of SAM children recovered from SAM, with a median time to recovery of 49 days (IQR: 35–63), or 7 weeks. The majority of the recoveries occurred within 4–6 weeks of starting OTP care, which is considered a longer stay for children under OTP care in comparison with the SPHERE minimum references. The majority of recoveries were experienced after 4 weeks of admission as indicated by the major increase in the number of recovered children between 4 and 7 weeks (Figure 1).

Upon Kaplan–Meir analysis, the recovery rate was substantially lower among children residing within >30 min of the health facility, on antibiotics, and non-breastfeeding children. Furthermore, there was a significant difference in survival time to recovery due to diarrhea, the presence of edema, and the absence of edema in children who took amoxicillin drugs as compared to those who were not on routine drugs. Using the log-rank test, we have checked for the significance of the observed differences in recovery rates among the categorical factors (Figure 1 and Table 4).

**Table 4:** Comparison of the median survival time to recovery from SAM by categorical predictors among children in eastern Ethiopia.

Variable	Options	Median survival time with 95% CI	X^2^	*p*-value of log-rank test
Admission weight category	<7 kg	62.3 (51.3–60.7)	42.1	0.0001
	>7 kg	47.5 (38.9–45.2)		
Distance from home to HP	< 30 min	49.0 (46.4–51.6)	12.8	0.0001
	> = 30 min	56.0 (50.4–61.6)		
Breastfeeding	Yes	56.0 (51.9–60.1)	47.8	0.0001
	No	42.0 (39.8–44.2)		
Type of SAM	Marasmus	56.0 (52.2–59.8)	30.0	0.0001
	Kwashiorkor	42.0 (38.7–45.3)		
Edema	Yes	42.0 (39.3–44.7)	42.4	0.0001
	No	56.0 (52.4–59.6)	30.0	
Diarrhea	Yes	56.0 (50.9–61.1)	11.9	0.001
	No	49.0 (46.1–51.9)		
Amoxicillin	Yes	49.0 (46.5–51.5)	29.2	0.0001
	No	63.0 (56.9–69.1)		
Deworming	Yes	42.0 (39.4–44.6)	14.2	0.0001
	No	56.0 (52.7–59.3)		
Edema	No	56.0 (52.4–59.6)	42.4	0.0001
	Yes	42.0 (39.3–44.7)		
Adequate RUTF	Not adequate	63.0 (56.8–69.3)	11.5	0.001
	Adequate	49.0 (46.5–51.5)		

The average time to recovery from SAM was relatively longer among children with lower admission weight (7 kg) and children coming from distant places from health posts (*p*-value of the log-rank test = 0.0001). Similarly, the time to recovery from SAM was significantly shorter among SAM children who were breastfed, had edema, received adequate RUTF based on their weight, took amoxicillin, and were dewormed as compared to their counterparts. Hence, children with these admission characteristics and treatment options had a better recovery time as compared to those without these attributes (Table 4).

### Predictors of time to recovery from uncomplicated SAM

We have checked the proportional hazard assumption before fitting the covariates into the regression model. Hence, all of the predictor variables did not violate the proportional hazards’ assumptions (*p* > 0.05), making the data suitable for the Cox proportional regression analysis. In the bivariable Cox regression model, the age of the child, MUAC and weight category, edema, taking amoxicillin, breastfeeding status, vaccination status, clinical forms of SAM, deworming, distance to a health facility, and having adequate RUTF were important predictors of cure from SAM in the crude model (*p* < 0.05).

In the adjusted model, distance from the OTP site to home, presence of edema and diarrhea on admission, breastfeeding status, source of referral, measles vaccination status, amoxicillin, and the adequacy of the prescribed RUTF amount were independent predictors of time-to-nutritional recovery from uncomplicated SAM. Children residing >30 min from a health post had a 44% higher probability of having a shorter recovery (AHR = 1.44; 95% CI: 1.13–1.85). In addition, children who took amoxicillin (AHR = 1.55; 95% CI: 1.19–2.02) and without measles vaccination [AHR = 1.46 (1.04–2.03)] were 55 and 46% more likely to have shorter recovery from SAM, respectively (Table 5).

**Table 5:** Bivariable and multivariable Cox regression output for factors determining time to recovery from SAM children at OTP in East Hararghe Zone, Ethiopia (*n* = 402).

Variable	Options	Treatment outcome		CHR 95% CI	AHR with 95% CI	*p*-value
		Cured	Censored			
Sex	Male	173	18	1.04 (0.845–1.279)		
	Female	187	24	1		
Age in months	<24	257	31	1.49 (1.18–1.88)*		
	24–59	103	11	1		
Referral system	Self-referred	121	13	1	1	
	Community volunteer and campaign	221	26	1.13 (0.90–1.41)	1.15 (0.90–1.45)	0.261
	From stabilization center and Other OTP	18	3	1.15 (0.70–1.89)	1.70 (1.02–2.84)	0.041
MUAC	<11.5	240	38	1		
	11.5–12.5	55	3	1.67 (1.24–2.24)*		
	>12.5	65	1	2.282 (1.73–3.01)*		
Admission weight category	<7 kg	170	33	1		
	>7 kg	190	9	1.85 (1.50–2.28)*		
Distance to health facility	<30 min	256	1	1.44 (1.15–1.81)*	1.44 (1.13–1.85)	0.004
	> = 30 min	104	41	1	1	
Breastfeeding	Yes	184	30	1	1	
	No	176	12	1.92 (1.56–2.38)**	1.60 (1.27–2.02)	0.000
Vaccination	Yes	235	25	1.15 (0.93–1.43)		
	No	125	17	1		
Type of SAM	Marasmus	239	36	1		
	Kwashiorkor	111	2	1.91 (1.5–2.40)**		
	Marasmic kwashiorkor	10	4	2.09 (1.10–3.96)*		
Diarrhea	Yes	111	37	1		
	No	249	5	1.42 (1.13–1.77)*	1.51 (1.18–1.94)	0.001
Amoxicillin	Yes	280	18	1.83 (1.42–2.35)*	1.55 (1.19–2.02)	0.001
	No	80	24	1	1	
Deworming	Yes	86	6	1		
	No	274	36	1.51 (1.19–1.93)*		
Measles	Yes	42	3	1		0.027
	No	318	39	1.15 (0.84–1.59)	1.46 (1.04–2.03)	
Chest indrawing	No	354	42	1.92 (0.84–4.37)		
	Yes	6	0	1		
Edema	No	239	36	1	1	
	Yes	121	6	1.92 (1.54–2.40)*	1.74 (1.33–2.29)	0.000
Admission type	New admission	350	40	1.49 (0.79–2.80)	1.62 (0.84–3.10)	0.148
	Readmission	10	2	1	1	
Adequate RUTF	Not adequate	55	9	1	1	
	Adequate	305	33	1.55 (1.16–2.07)*	1.22 (0.90–1.65)	0.206

The asterisks indicate statistically significant predictors of time to recovery from SAM at a *p*-value below 0.05 (*) and 0.001 (**).

The time to recovery was better among children without diarrhea (AHR = 1.51; 95% CI: 1.18–1.94), with edema (AHR = 1.74; 95% CI: 1.33–2.29) at admission, and new admissions to the OTP (AHR = 1.62; 95% CI: 0.84–3.91) than counterparts. Self-referred children had a significantly longer time to recover from uncomplicated SAM as compared to those referred from inpatient care or community outreach. Furthermore, the time to recovery was significantly shorter for children diagnosed with marasmic kwashiorkor and kwashiorkor than those children with marasmus. More importantly, administering adequate RUTF in accordance with the SAM protocol could improve the time to recovery (AHR = 1.22; 95% CI: 0.90–1.65) (Table 5).

## Discussion

The current study was to identify the potential factors predicting time to recovery from SAM in low-income settings, in eastern Ethiopia. The findings of this study showed that 89.6, 9.5, 0.7, and 0.2% were recovered, defaulted, dead, and non-respondents, respectively. All indicators met the minimum performance indicator and were within the Sphere Standard range, which is above 75% (21). The Sphere Standards are a set of guidelines and principles for humanitarian response. It establishes minimum standards and principles in areas such as water, sanitation, shelter, health, and protection, with a focus on promoting accountability and the wellbeing of affected populations. Adhering to these standards helps ensure effective and coordinated humanitarian assistance while prioritizing the rights and dignity of those in need (21).

On the other hand, the average length of stay within the program was in an alarming range, where a child could stay for more than 7 weeks. The current study showed a better cure rate than studies conducted in different parts of the globe, which range from 10.2 to 77.9% (30). Comparable cure rates were reported from Afar (83.2%) (31), southern Ethiopia (85%) (25), and Tigray (87%) (32), which are above the Sphere Standards. However, due to the curable nature of SAM, expanded interventions in the home environment and care at OTP sites could improve timely recovery. Through this, full recovery can be achieved by reducing a significant number of child deaths. It should also be noted that the Sphere Standards are mainly for humanitarian settings, where basic livelihoods are severely affected.

Both the death and default rates were within the acceptable range of the International Sphere Humanitarian Standard reference, which is 10 and 15%, respectively (21). The study also showed a default rate of 9.5%, which is below the standard reference point and lower than studies conducted in Yemen, where 80.2% of SAM had defaulted and only 10.2% had recovered (33), which could be related to the emerging disasters and poor service delivery in Yemen. It is worse than studies conducted in the Afar region (6.3%) (25), the Tigray region (2.2%) (34), and Kenya (2.9%) (32). The reported death rate in our study is lower than studies from the Afar region (4.9%) (33), and Yemen (3.3%) (32). In this study, the median length of stay at the OTP site was 7 weeks, considered alarming and not acceptable compared to the minimum international standard of 4 weeks, yet it is acceptable based on the national SAM protocol (16 weeks) (23). It is also consistent with other studies in southern Ethiopia showing 6.7–8.7 weeks (25, 35, 36), which is lower than previous studies (9–10 weeks) (7, 32, 33). These discrepancies could be linked to service quality, adherence to care, the practice of sharing RUTF, suitable home environments, and the severity of SAM at admission.

Children coming from a nearby health post (less than 30 min) had 44% higher odds of having rapid recovery from SAM as compared to children from farther locations. A study from the Kamba district showed that residents from distant areas could affect recovery from SAM, but it was not statistically significant (35). These could be due to poor physical access to weekly OTP follow-up and poor treatment adherence as compared to those from near the facility.

Children who received amoxicillin therapy had a 55% higher probability of recovery than their counterparts. The finding is consistent with the finding from Tigray Region (20) and clinical trials in antibiotics are a crucial part of SAM treatment in India (37), which had the potential to reverse hidden infections such as pneumonia or bowel bacterial overgrowth (23). On the other hand, those children who did not get measles at admission usually had the vaccination already, making them protected.

More importantly, time to recovery was better for children with edema, including kwashiorkor and marasmic kwashiorkor. As opposed to the fact that edematous malnutrition is associated with many complications and is usually treated in inpatient care, these children might have been referred to a stabilization center (SC) or have a better nutritional index. These would shorten the recovery period. Previous studies conducted in Ethiopia (25, 32) and Ghana (38) showed that children admitted with kwashiorkor had a rapid recovery compared to marasmic children. Another study from Ethiopia did not indicate these associations (35). This could be explained by the concurrent presence of other medical morbidities that would reduce recovery and provide better care for kwashiorkor children (19). In contrast, OTP implementation is challenged by many factors, including the sharing of RUTF among family members (2, 39), which is usually associated with poor knowledge and limited household food access (40). Thus, the perceived severity of kwashiorkor might influence caregivers’ decisions not to share the RUTF (41).

Medical comorbidities were associated with a longer recovery from SAM, specifically the presence of diarrhea. The likelihood of timely recovery was 51% higher among those without diarrhea. This finding was consistent with a study conducted in the southern part of Ethiopia (26) and northern Ethiopia (7). This could be explained by the vicious cycle of diarrhea and SAM affecting recovery time mainly due to metabolic disturbances, fluid and electrolyte losses, and dehydration (5). This evidence is also in line with the fact that a multitude of studies implied diarrhea as a major determinant predicting time to recovery from uncomplicated SAM (32, 42).

Those children who were not breastfed have limited options for food and are more likely to consume the prescribed RUTF, leading to rapid recovery. Due to overreliance on breastfeeding, adherence to RUTF might be limited, increasing the risk of sharing. This scenario can occur when infants are less likely to take the full dose of RUTF as it may be less palatable and have less acceptable textures compared to breast milk. Furthermore, sharing RUTF among household members could reduce cure rates (7) and average weight gain (43). Arguably, sharing of RUTF is more likely with older siblings of a breastfeeding infant than their counterparts because the mother can provide better nutritional care to the affected child along with breast milk. Hence, strong adherence counseling should be in place for those who on breastfeed although breastfeeding is growth-promoting for children.

While a few studies depicting the link between breast-feeding and time to recovery from SAM found none (44, 45); however, neither study did a sub-analysis for children under 2 years or explored the link between breast-feeding and improvement in MUAC. The small weekly increase in MUAC among breast-feeding children below 2 years of age was unexpected and needs careful consideration. Beyond the first 6 months, breastfeeding without adequate complementary feeding is inadequate for meeting the growing needs of children (46). Hence, providing RUTF serves to improve recovery from SAM as a short-term intervention.

Those children who get adequate RUTF according to their weight have a better time recovering from SAM. A study showed that a reduced dose of RUTF could significantly reduce weight, which was pronounced among infants under 1 year (47). In addition, alternative ready-to-use foods were found to be inferior to the standard RUTF in Ghana (48).

The study provides valuable insights into the context-specific predictors of severe acute malnutrition (SAM) and the effectiveness of management practices. It highlights the potential benefits of utilizing extended packages for managing uncomplicated SAM through trained community health workers or health extension workers (49, 50). This approach has shown promise in reducing the adverse impacts of SAM at an early stage as demonstrated by previous studies (8, 22, 51). By leveraging the expertise and accessibility of community-based healthcare providers, there is an opportunity to improve the timeliness and effectiveness of interventions, ultimately leading to better outcomes for individuals affected by SAM. These findings contribute to the existing body of knowledge and support the importance of community-based approaches in addressing SAM effectively.

## Limitations of the study

Although this study generated valuable evidence, data on RUTF sharing practices, selling behaviors, and food security status are frequently reported as risk factors for recovery (51). However, due to the retrospective design of the study, these pieces of data were not captured, limiting the interpretation of this study. Moreover, the presence of some missing data could also further limit the conclusions derived from this study. The lack of data on poverty or family income due to the secondary nature of the data could limit the interpretability of the study findings.

## Conclusion

Overall, the recovery rate from SAM among children on OTP was found to be optimal in accordance with the Sphere Standards, yet there is a need for further improvements. In addition, this is below the universal target to cure all children from SAM. The proximity of the health posts to the patient’s house, the occurrence of diarrhea, the kind of malnutrition, and the availability of amoxicillin were all associated with time to recovery. Comorbid diseases must also be properly treated in order to improve the cure rate. Health extension workers counsel the mother if the child is breastfeeding, offer breast milk on demand, and feed RUTF according to the guidelines, but need to follow up with the child to ensure the prescribed RUTF is consumed properly. Enhanced health education packages and community engagement could improve early recovery. Prospective studies quantifying the food security situation and RUTF-sharing practices could generate valuable evidence for targeted interventions. Therefore, the conclusions of the study may help current SAM treatments identify high-risk kids who have a bad prognosis and target them with additional care and therapies.

## Data availability statement

The original contributions presented in the study are included in the article/supplementary material, further inquiries can be directed to the corresponding author.

## Ethics statement

The studies involving humans were approved by Addis Ababa Medical and Business College. The studies were conducted in accordance with the local legislation and institutional requirements. The ethics committee/institutional review board waived the requirement of written informed consent for participation from the participants or the participants’ legal guardians/next of kin because it is secondary data review where informed consent was not applicable.

## Author contributions

SY: Conceptualization, Data curation, Formal analysis, Funding acquisition, Investigation, Methodology, Project administration, Resources, Software, Supervision, Writing – original draft, Writing – review & editing. TT: Conceptualization, Investigation, Methodology, Project administration, Resources, Supervision, Validation, Writing – review & editing. TN: Conceptualization, Data curation, Investigation, Methodology, Project administration, Resources, Writing – original draft, Writing – review & editing. BH: Conceptualization, Investigation, Methodology, Resources, Writing – review & editing. AK: Conceptualization, Investigation, Supervision, Validation, Writing – review & editing. AM: Investigation, Methodology, Resources, Validation, Writing – review & editing. DA: Data curation, Investigation, Resources, Software, Writing – review & editing. AO: Conceptualization, Data curation, Formal analysis, Investigation, Methodology, Project administration, Resources, Software, Visualization, Writing – original draft, Writing – review & editing. KR: Conceptualization, Data curation, Investigation, Methodology, Resources, Supervision, Validation, Writing – review & editing.
